# Diabetes free life expectancy and years of life lost associated with type 2 diabetes: projected trends in Germany between 2015 and 2040

**DOI:** 10.1186/s12963-021-00266-z

**Published:** 2021-10-11

**Authors:** Thaddäus Tönnies, Jens Baumert, Christin Heidemann, Elena von der Lippe, Ralph Brinks, Annika Hoyer

**Affiliations:** 1 Institute for Biometrics and Epidemiology, German Diabetes Center (DDZ), Leibniz Center for Diabetes Research at Heinrich Heine University, Düsseldorf, Germany; 2 Department of Epidemiology and Health Monitoring, Robert Koch Institute, Berlin, Germany; 3 Chair for Medical Biometry and Epidemiology, Faculty of Health/School of Medicine, Witten/Herdecke University, Witten, Germany; 4 Department of Statistics, Ludwig Maximilians University Munich, Munich, Germany

**Keywords:** Type 2 diabetes, Years of life lost, Burden of disease, Healthy life years, Projection, Mathematical model, Illness-death model

## Abstract

**Background:**

Type 2 diabetes (T2D) causes substantial disease burden and is projected to affect an increasing number of people in coming decades. This study provides projected estimates of life years free of type 2 diabetes (T2D) and years of life lost (${\mathrm{YLL}}$) associated with T2D for Germany in the years 2015 and 2040.

**Methods:**

Based on an illness-death model and the associated mathematical relation between prevalence, incidence and mortality, we projected the prevalence of diagnosed T2D using currently available data on the incidence rate of diagnosed T2D and mortality rates of people with and without diagnosed T2D. Projection of prevalence was achieved by integration of a partial differential equation, which governs the illness-death model. These projected parameters were used as input values to calculate life years free of T2D and ${\mathrm{YLL}}$ associated with T2D for the German population aged 40 to 100 years in the years 2015 and 2040, while accounting for different assumptions on future trends in T2D incidence and mortality.

**Results:**

Assuming a constant incidence rate, women and men at age 40 years in 2015 will live approximately 38 years and 33 years free of T2D, respectively. Up to the year 2040, these numbers are projected to increase by 1.0 years and 1.3 years. Assuming a decrease in T2D-associated excess mortality of 2% per year, women and men aged 40 years with T2D in 2015 will be expected to lose 1.6 and 2.7 years of life, respectively, compared to a same aged person without T2D. In 2040, these numbers would reduce by approximately 0.9 years and 1.6 years. This translates to 10.8 million and 6.4 million ${\mathrm{YLL}}$ in the German population aged 40–100 years with prevalent T2D in 2015 and 2040, respectively.

**Conclusions:**

Given expected trends in mortality and no increase in T2D incidence, the burden due to premature mortality associated with T2D will decrease on the individual as well as on the population level. In addition, the expected lifetime without T2D is likely to increase. However, these trends strongly depend on future improvements of excess mortality associated with T2D and future incidence of T2D, which should motivate increased efforts of primary and tertiary prevention.

**Supplementary Information:**

The online version contains supplementary material available at 10.1186/s12963-021-00266-z.

## Background

The prevalence of diabetes has increased globally and considering all types of diabetes, it is estimated that there were 415 million cases in 2015 in the population aged 20–79 years [1]. Due to increased mortality and morbidity, diabetes is one of the leading causes of disease burden in many countries [2]. Commonly, disease burden due to increased mortality is quantified by years of life lost due to premature death (${\mathrm{YLL}}$). ${\mathrm{YLLs}}$ inform about the average life time a person loses through premature death from a certain disease.

T2D as an underlying cause of death still imposes a large disease burden in Germany, however trends indicate that the burden is shifting towards increased morbidity [3]. This is partly caused by reductions in the mortality rate among people with T2D in the last decades [4–6]. Hence, the average life time spent with T2D for the individual and in the population is likely to increase [6, 7]. Thus, besides ${\mathrm{YLL}}$, quantifying lifetime free of T2D (T2D free life expectancy), is one way to give a more complete picture of the burden T2D imposes on a population.

Whilst there are several approaches to estimate ${\mathrm{YLL}}$ [8, 9], the global burden of disease study (GBD) probably is the most widely known application of the concept [2]. In GBD, ${\mathrm{YLL}}$ are based on the number and causes of deaths in a given year and the expected remaining life expectancy at the age of death. For instance, it was estimated that in Germany, 256,217 years of life were lost due to diabetes as cause of death in 2017, ranking diabetes at the 13th position in the causes of ${\mathrm{YLL}}$ [10]. The GBD estimates are based on a global standard life expectancy. Although using a standard life expectancy is well suited for between country comparisons, estimates based on country-specific life expectancy might be more appropriate to inform national policies. For T2D in particular, there are two methodological specificities with the ${\mathrm{YLL}}$ method used in GBD. First, causes of death statistics probably underestimate the number of deaths due to T2D [11] as comorbidities and complications of diabetes (e.g. cardiovascular diseases), which are highly prevalent in people with diabetes and common causes of death, are not taken into account by this approach. Second, ${\mathrm{YLL}}$ based on observed number of deaths does not consider lower life expectancy of people with T2D. In contrast, methods based on excess mortality rates associated with T2D (as in [9]) are another approach, which compare people with T2D to people without T2D (including comorbidities) with regard to life expectancy. This approach might be preferable when the aim is to describe the mortality burden associated with prevalent T2D, as opposed to the mortality burden among people dying in a given year with type 2 diabetes as the documented cause of death.

Previous studies estimating ${\mathrm{YLL}}$ associated with prevalent T2D relied on cross-sectional mortality rates, for instance from period life tables [e.g. 12, 13]. However, life expectancy of people alive today depends on mortality rates these people will experience in the future. Therefore, accounting for future trends in mortality rates rather than assuming currently observed mortality rates may yield more valid estimations.

Hence, this study aims to provide estimates of ${\mathrm{YLL}}$ associated with diagnosed T2D and T2D-free life expectancy for Germany that overcome these concerns. We use a mathematical model to estimate the burden associated with T2D in 2015 and project temporal trends up to 2040.

## Methods

### Study design

For this study, we considered the German population aged between 40 and 100 years in the years 2015 and 2040. We defined the lower age limit of 40 years, because T2D prevalence at younger ages is relatively low [14]. The upper age limit was chosen based on the upper age limit in official population statistics [15].

In order to estimate T2D-free life expectancy and ${\mathrm{YLL}}$ at age 40 in 2015 and 2040 and to account for future trends in mortality and incidence rates, projections of these rates and the resulting prevalence of T2D were needed. The year 2100 was the upper bound of the projections in this setting, because a person aged 40 years in 2040 would be 100 years in 2100. Hence, in order to estimate T2D-free life expectancy and ${\mathrm{YLL}}$ associated with T2D for this person, the projected rates and prevalence between 2040 and 2100 were needed.

We used the mathematical model presented in the following section to project the age-specific incidence rate, mortality rates of people with and without T2D and the prevalence between 2015 and 2040. The projected parameters serve as input values to calculate T2D-free life expectancy and ${\mathrm{YLL}}$ associated with T2D. All analyses were stratified by sex.

### Mathematical model

The projection was based on the illness-death model. In this model, people can be in one of the three states “healthy”,”ill” and “dead”. In the current case, healthy and ill referred to the absence and presence of diagnosed T2D. Transitioning between states is expressed in terms of age and calendar time specific rates $i$ (incidence rate from healthy to ill), ${m}_{0}$ (mortality rate in the healthy state) and ${m}_{1}$ (mortality rate in the ill state). The proportion of the population in the ill state is the prevalence $p$. All parameters of the illness-death model depend on calendar time $t$ and age $a$. It has been shown that this system is governed by a partial differential equation [16, 17]:

1 $${(\partial }_{t}{+\partial }_{a})p=\left(1-p\right)\cdot \left[i-m\cdot \frac{p\cdot \left({\mathrm{MRR}}-1\right)}{p\cdot \left({\mathrm{MRR}}-1\right)+1}\right]$$

where ${(\partial }_{t}{+\partial }_{a})p$ is the temporal change of the age-specific prevalence, $m$ is the mortality rate of the general population, and ${\mathrm{MRR}}$ is the mortality rate ratio for people with T2D versus without T2D (${m}_{1}$/${m}_{0}$).

We solved Eq. (1) by integration using input values for the incidence rate, general mortality rate and ${\mathrm{MRR}}$, to project the age-specific prevalence up to the year 2100.

### Data sources

The projections were based on the age-specific prevalence and incidence rate of T2D observed in 2010 among all people aged 40–100 years in the German statutory health insurance (*N* ≈ 65 million) [14]. People with prevalent T2D were identified by the corresponding diagnostic codes of the International Classification of Diseases (E11) documented in inpatient or outpatient care facilities. Prevalence of T2D among men and women aged ≥ 40 years was estimated with 7.4% and 7.0%, respectively (see Additional file 1 for age-specific prevalence). The incidence rate was derived from a mathematical model that uses information on T2D prevalence, general mortality and ${\mathrm{MRR}}$ [14]. For men and women aged ≥ 40 years, the incidence rates were 16 and 13 cases per 1000 person years, respectively. Further details can be found in Tamayo et al. [14] and in Additional file 1. Information on the age-specific diabetes-related ${\mathrm{MRR}}$ was based on the same data, but from the years 2013/2014 [18]. Schmidt et al. [18] estimated the ${\mathrm{MRR}}$ comparing 6.5 million people with and 40.8 million people without diabetes (all types) in 2013 with regard to their vital status in 2014. The MRR ranged between 6.9 among men aged 30–34 years and 1.1 among men aged ≥ 95 years (further details in Additional file 1). Although the ${\mathrm{MRR}}$ was not differentiated by diabetes type, these data serve as a reasonable approximation for the ${\mathrm{MRR}}$ associated with T2D, since 90% of cases are estimated to be of this type and the incidence rate of type 1 diabetes is highest in people younger than 20 years [19, 20]. The age-specific all-cause mortality rate in the general population ($m$) between 2010 and 2040 stems on the population projections of the Federal Statistical Office which is based on population statistics dating back to 1871 (Additional file 1) [15]. Based on this data from 2010, we projected the age-specific prevalence of T2D up to the year 2100 and calculated T2D-free life expectancy and ${\mathrm{YLL}}$ in 2015 and 2040. Further information on how Eq. (1) can be used for projections is available in [21].

For each year beyond 2010, input values were chosen based on assumptions on temporal trends in the T2D incidence rate and the ${\mathrm{MRR}}$. As base case, we chose a constant incidence rate, since long-term trends in the age-specific incidence rate are not available in Germany [22]. For the ${\mathrm{MRR}}$, we assumed an annual decrease of 2%, since there is consistent evidence from several countries that the ${\mathrm{MRR}}$ is decreasing with calendar time [4, 5, 23]. However, since it seems unlikely that the difference in mortality between people with and without T2D will disappear completely, we assumed a lower limit of 1.1 for the ${\mathrm{MRR}}$. In addition to the base case, we assumed three alternative scenarios (A, B and C) as depicted in Table 1. We chose these scenarios to account for uncertainty in the outcomes due to unknown future trends and to assess the impact of different trends in the incidence rate and the ${\mathrm{MRR}}$ on the outcomes. Since, the uncertainty in the results due to sampling error is very small compared to the variability between the different projection scenarios, we focus on the variability due to unknown trends in incidence and mortality rates and do not report confidence intervals. In Additional file 2, we show that the sampling error is negligible compared to the variability between the scenarios. Another reason to omit confidence intervals is that the input data almost comprises the whole population (approximately 90%). Hence, inferential statistics to generalize from a sample to the population might not be necessary in this case.

**Table 1:** Scenarios of the prevalence projections

Scenario	Annual change in …	
	Incidence rate	Mortality rate ratio
Base case	0	− 2%
A	0	0
B	− 0.5%	− 2%
C	+ 0.5%	− 2%

Assumed temporal trends in incidence and mortality between 2015 and 2040

### Calculation of T2D-free life expectancy

We used the method of Sullivan [24] to estimate T2D-free life expectancy up to age 100:

2 $${\mathrm{T2D}}\, {\mathrm{free}}\, {\mathrm{life}}\, {\mathrm{expectancy}}\left(t,a\right)=\frac{1}{S\left(t,a\right)}\underset{0}{\overset{\infty }{\int }}\left(1-p\left(t+u,a+u\right)\right)S\left(t+u,a+u\right){\mathrm{d}}u$$

where $S$ is the survival function of the general population based on projections of the mortality rate from the Federal Statistical Office (details in Additional file 3). For $p$, we used the projected prevalence from Eq. (1). T2D-free life expectancy in a given year $t$ was approximated by multiplying the probability of being free of T2D ($1-p$) with the probability of being alive ($S$) for each year between age $a=40$ and $a=100$. The integral of this product yields the T2D-free life expectancy at age 40 years.

### Calculation of years of life lost associated with type 2 diabetes

Similar to previous studies [9, 25, 26], we estimated ${\mathrm{YLL}}$ as the difference in life expectancy between a person with T2D and a person of the same age without T2D. This approach is formally defined by the following equation:

3 $${\mathrm{YLL}}\left(t,a\right)=\underset{0}{\overset{\infty }{\int }}{S}_{D-}\left(t+u,a+u\right)-{S}_{D+}\left(t+u,a+u\right){\mathrm{d}}u$$

where $t$ and $a$ are calendar time and age, respectively. ${S}_{D-}$ and ${S}_{D+}$ are the survival functions of people without T2D and with T2D, respectively. These survival functions were based on projections of the mortality rate $m$ of the general population as reported by the Federal Statistical Office [15] and on the ${\mathrm{MRR}}$ reported by Schmidt et al. [18] to yield diabetes-specific mortality rates ${m}_{1}$ and ${m}_{0}$ (details in Additional file 3). For the calculation of ${\mathrm{YLL}}$, we assumed that a person without T2D would not develop T2D after time $t$.

Henceforth, we refer to Eq. (3) as individual ${\mathrm{YLL}}$, since it represents the comparison of two persons of the same birth cohort at a certain calendar time. As a population-wide measure of ${\mathrm{YLL}}$, we calculated population-wide ${\mathrm{YLL}}$ by summing up individual ${\mathrm{YLL}}$ over the age distribution in the population at time $t$. Population-wide ${\mathrm{YLL}}$ can be interpreted as the ${\mathrm{YLLs}}$ among those with prevalent T2D at time $t$. This population-wide ${\mathrm{YLL}}$ is not comparable to the GBD approach, which sums up the remaining life expectancies of people dying with T2D as the documented cause of death in death certificates. Since there are more people with prevalent T2D than people dying with T2D as the documented cause of death, it is expected that our approach results in more ${\mathrm{YLL}}$ compared to the GBD approach.

## Results

### T2D-free life expectancy

**Fig. 1:**
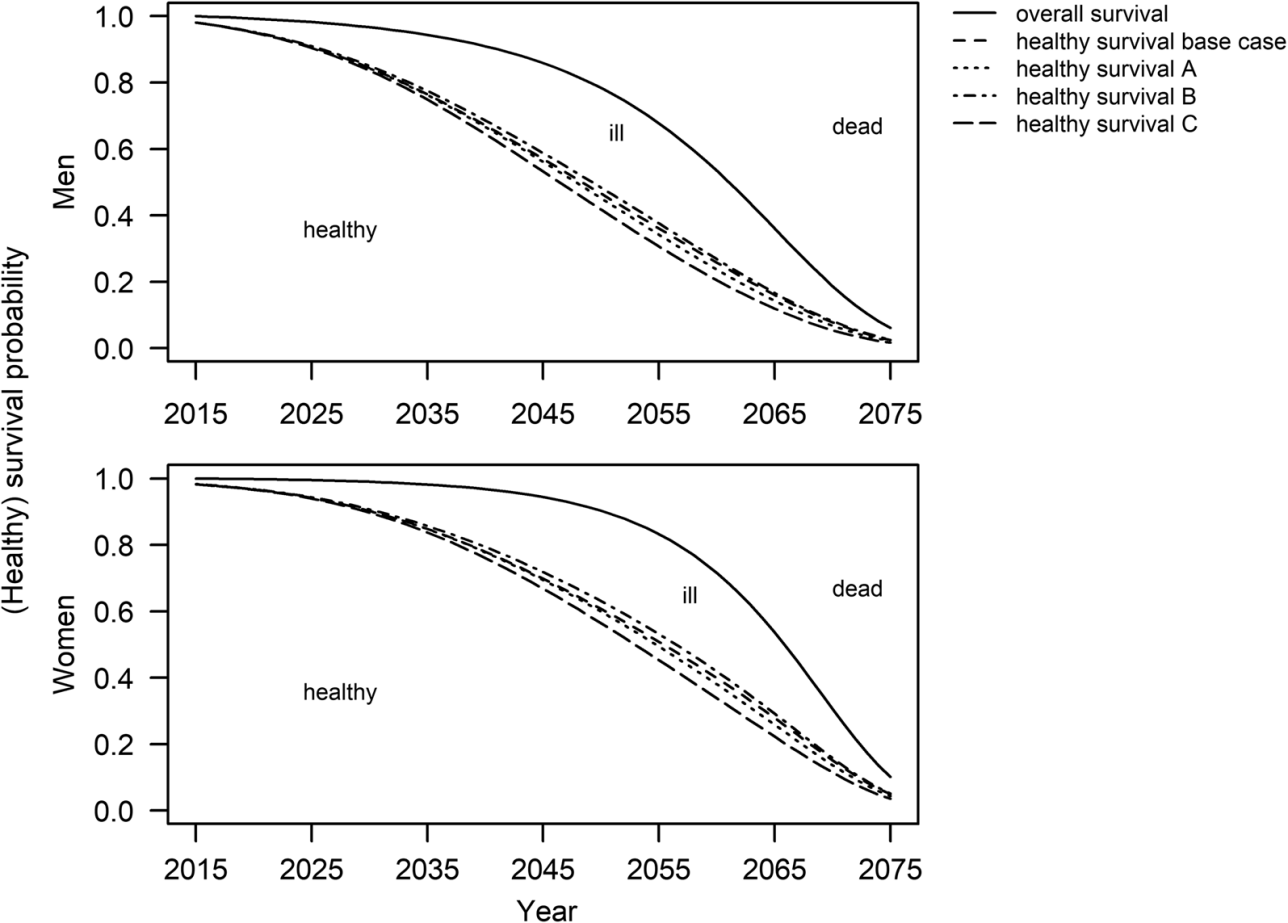
Survival and healthy survival probability of men and women aged 40 years in 2015. Different line patterns indicate different scenarios as depicted in Table 1. At each age, the healthy survival probability indicates the probability of being alive and free of type 2 diabetes. Base case: $i$ constant, ${\mathrm{MRR}}$ − 2%; A: $i$ constant, ${\mathrm{MRR}}$ constant; B: $i$ − 0.5%, ${\mathrm{MRR}}$ − 2%; C: $i$ + 0.5%, ${\mathrm{MRR}}$ − 2%

Figure 1 illustrates the survival probability for a person aged 40 years in 2015 up to age 100 in 2075, based on the projected mortality rate of the Federal Statistical Office. In addition, the figure illustrates the probability of being alive and free of T2D (“healthy”) for this person. Hence, for people in 2015 aged 40 years, one can infer the probability of being in the state “ill”, “healthy” or “dead” at each time point between 2015 and 2075. At age 100, the probability of being alive and healthy is very close to zero in all scenarios. Thus, we assume ages over 100 years to be negligible for this study.

In Fig. 1, each of the three states of the illness-death model is designated by a corresponding area. Thus, the area below the healthy survival curve are the years spent in the “healthy” state, hence the T2D-free life expectancy, which were calculated using the formula by Sullivan [24]. It follows that the area between the overall survival curve and the healthy survival curve represents years spent in the “ill” state. The sum of T2D-free life expectancy and years spent in the “ill” state is the total life expectancy, which is equal to the area under the overall survival curve.

Table 2 shows the T2D-free life expectancy at age 40 years for different scenarios in 2015 and 2040. The T2D-free life expectancy in 2015 for the base case were 38.1 years and 32.7 years for women and men, respectively. In 2040, T2D-free life expectancy is projected with 39.1 years (absolute change: + 1.0 years) and 34.0 years (+ 1.3 years) among women and men, respectively. The base case assumed annual decreases of the ${\mathrm{MRR}}$ by 2%. Comparing the results to a scenario with constant ${\mathrm{MRR}}$ (scenario A), shows that trends in ${\mathrm{MRR}}$ only had minor influence on T2D-free life expectancy. In contrast, minor annual changes in the incidence rate of ± 0.5% (scenarios B and C) had a comparably large impact on T2D-free life expectancy in 2040. Decreases in the incidence rate resulted in an increase of T2D-free life expectancy by 2.2 years and 2.7 years among women and men, respectively. In contrast, increases in the incidence rate resulted in a decrease in T2D-free life expectancy by 0.5 years and 0.1 years among women and men, respectively.

**Table 2:** Projected life expectancy free of type 2 diabetes (T2D-free LE) in 2015 and 2040

Scenario	T2D-free LE in 2015 (in years)	T2D-free LE in 2040 (in years)	Absolute change (in years)	Relative change (in percent)
*Women*				
Base case^#^	38.1	39.1	1.0	2.6
A	38.5	39.5	1.0	2.6
B	39.2	41.4	2.2	5.6
C	36.9	36.4	− 0.5	− 1.4
*Men*				
Base case^#^	32.7	34.0	1.3	4.0
A	33.2	34.5	1.3	3.9
B	33.7	36.4	2.7	8.0
C	31.6	31.5	− 0.1	− 0.3

T2D-free LE refers to age 40

Scenarios assumed different annual trends in incidence rate ($i$) and mortality rate ratio (${\mathrm{MRR}}$) associated with diabetes: Base case: $i$ constant, ${\mathrm{MRR}}$ − 2%; A: $i$ constant, ${\mathrm{MRR}}$ constant; B: $i$ −  0.5%, ${\mathrm{MRR}}$ − 2%; C: $i$ + 0.5%, ${\mathrm{MRR}}$ − 2%

### Years of life lost associated with type 2 diabetes

**Fig. 2:**
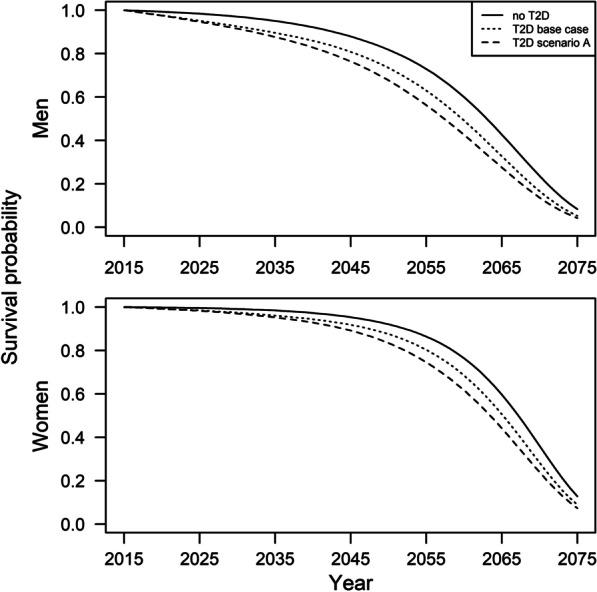
Survival probability at age 40 years in 2015 with and without type 2 diabetes (T2D). The survival functions for people with T2D in scenarios B and C in Table 1 were identical to the base case scenario. Base case: $i$ constant, ${\mathrm{MRR}}$ − 2%; A: $i$ constant, ${\mathrm{MRR}}$ constant; B: $i$ − 0.5%, ${\mathrm{MRR}}$ − 2%; C: $i$ + 0.5%, ${\mathrm{MRR}}$ − 2%

Figure 2 illustrates the survival curves for people with and without T2D with regard to different assumptions on the incidence rate and ${\mathrm{MRR}}$. In the base case as well as scenarios B and C we assumed a decreasing ${\mathrm{MRR}}$. In these scenarios people with T2D had a considerably higher survival probability compared to assuming constant ${\mathrm{MRR}}$ (scenario A). Contrary, temporal trends of the incidence rate (scenario B and C) had almost no impact on survival of the diabetic population.

The areas between the curves for no T2D and the curves for the T2D scenarios represents the ${\mathrm{YLL}}$ for a person aged 40 years in 2015 with T2D compared to a person aged 40 years in 2015 without T2D. Hence, one can see from Fig. 2 that changes in ${\mathrm{MRR}}$ have a considerable impact on ${\mathrm{YLL}}$.

We calculated this area for each scenario in 2015 and 2040 using Eq. (3) (Table 3). In the base case, men and women with T2D aged 40 years in 2015 lost 2.7 and 1.6 years, respectively, compared to a same aged person without T2D. In 2040, the results indicate a substantial decrease in ${\mathrm{YLL}}$ by 0.9 years and 1.6 years among women and men, respectively. Assuming constant instead of decreasing ${\mathrm{MRR}}$ (scenario A) resulted in more than twice as many ${\mathrm{YLLs}}$ compared to the base case scenario in 2015. Also the decrease of ${\mathrm{YLL}}$ in 2040 was much weaker.

**Table 3:** Years of life lost (YLL) associated with type 2 diabetes on the individual level

Scenario	YLL in 2015 (in years)	YLL in 2040 (in years)	Absolute change (in years)	Relative change (in percent)
*Women*				
Base case	1.6	0.7	− 0.9	− 56.3
A	4.2	3.1	− 1.1	− 26.2
B	1.6	0.7	− 0.9	− 56.3
C	1.6	0.7	− 0.9	− 56.3
*Men*				
Base case	2.7	1.1	− 1.6	− 59.3
A	5.8	4.5	− 1.2	− 22.4
B	2.7	1.1	− 1.6	− 59.3
C	2.7	1.1	− 1.6	− 59.3

YLL refer to age 40 years on in the year 2015 and 2040.

Scenarios assumed different annual trends in incidence rate ($i$) and mortality rate ratio (${\mathrm{MRR}}$) associated with diabetes: Base case: $i$ constant, ${\mathrm{MRR}}$ − 2%; A: $i$ constant, ${\mathrm{MRR}}$ constant; B: $i$ − 0.5%, ${\mathrm{MRR}}$ − 2%; C: $i$ + 0.5%, ${\mathrm{MRR}}$ − 2%

We summed up age-specific individual ${\mathrm{YLL}}$ for people aged 40 to 100 years to calculate population-wide ${\mathrm{YLL}}$ (Table 4). Overall, ${\mathrm{YLL}}$ decreased from 10.8 million in 2015 to 6.4 million (− 4.4 million years) in 2040 in the base case scenario. In contrast to the base case scenario, assuming constant ${\mathrm{MRR}}$ increased population-wide ${\mathrm{YLL}}$ by 4.1 million years. Unlike individual ${\mathrm{YLL}}$, population-wide ${\mathrm{YLL}}$ in 2040 are strongly affected by trends in the incidence rate (scenario B and C).

**Table 4:** Years of life lost (YLL) associated with type 2 diabetes on the population level

Scenario	YLL in 2015 (in million years)	YLL in 2040 (in million years)	Absolute change (in million years)	Relative change (in percent)
*Women*				
Base case	4.87	2.77	− 2.10	− 43.2
A	8.33	10.11	1.78	21.3
B	4.85	2.56	− 2.29	− 47.2
C	4.89	2.99	− 1.90	− 38.3
*Men*				
Base case	5.88	3.58	− 2.30	− 39.1
A	10.28	12.63	2.35	22.9
B	5.86	3.31	− 2.54	− 43.4
C	5.91	3.87	− 2.04	− 34.5
*Overall*				
Base case	10.75	6.35	− 4.40	− 41.0
A	18.61	22.74	4.13	22.2
B	10.70	5.87	− 4.83	− 45.1
C	10.80	6.86	− 3.94	− 36.5

Scenarios assumed different annual trends in incidence rate ($i$) and mortality rate ratio (${\mathrm{MRR}}$) associated with diabetes: Base case: $i$ constant, ${\mathrm{MRR}}$ − 2%; A: $i$ constant, ${\mathrm{MRR}}$ constant; B: $i$ − 0.5%, ${\mathrm{MRR}}$ − 2%; C: $i$ + 0.5%, ${\mathrm{MRR}}$ − 2%

## Discussion

### Main findings

Assuming a constant incidence rate, we found that women and men at age 40 years in 2015 will live approximately 38 years and 33 years free of diagnosed T2D, respectively. Up to the year 2040, these numbers are projected to increase by 1.0 years and 1.3 years. However, we also found that small annual changes in future incidence rates strongly influence these trends. For instance, an annual increase in the incidence rate of 0.5% would result in decreases of T2D-free life expectancy by − 0.5 years and − 0.1 years among women and men, respectively, whilst an analogous decrease results in 2.2 years and 2.7 years increase in T2D-free life expectancy. Trends in ${\mathrm{MRR}}$ had no relevant impact on T2D-free life expectancy.

Assuming a continued decrease in ${\mathrm{MRR}}$ of 2% per year, we found that women and men aged 40 years with diagnosed T2D in 2015 lose 1.6 and 2.7 years of life, respectively, compared to a same aged person without T2D. In 2040, these numbers would reduce by approximately 0.9 years and 1.6 years. These reductions will be much smaller, if excess mortality does not improve after 2015. Trends in T2D incidence had no relevant impact on individual ${\mathrm{YLL}}$ associated with T2D. In the whole German population aged ≥ 40 years, ${\mathrm{YLL}}$ associated with T2D amounted to 10.8 million years in 2015 in case of an annual decrease in ${\mathrm{MRR}}$ of 2%. In 2040, this number will decrease by 4.4 million years. Depending on annual increases or decreases in the incidence rate of 0.5%, population-wide ${\mathrm{YLL}}$ will decrease by 3.9 million years or 4.8 million years, respectively. In case of no further improvements in ${\mathrm{MRR}}$, population-wide ${\mathrm{YLL}}$ would strongly increase to 18.6 million years in 2015 and further increase by 4.1 million years until 2040. In this scenario, ${\mathrm{YLL}}$ were already higher in 2015, because people with T2D would experience the higher ${\mathrm{MRR}}$ beyond 2015, in contrast to decreasing ${\mathrm{MRR}}$ in the other scenarios.

### Comparison to previous studies

In general, some previous studies estimated lower T2D-free life expectancy than we did. For instance, a regional German study estimated T2D-free life expectancy at age 40 years in 2014 with 34 years and 29 years for women and men, respectively [12], compared to 38 years and 33 years in our study. Cunningham et al. [13] reported T2D-free life expectancy of 30 years and 33 years at age 40 years in 2004 for men and women in the U.S. In Australia, men and women aged 45 years between 2000 and 2005 were estimated to live 30 and 34 years without T2D [27]. However, direct comparisons of these results are problematic, since most studies relied on cross-sectional mortality rates and (implicitly) assumed that these remain constant beyond the study period. In contrast, we used projected prevalence and simultaneously accounted for projected decreases in general mortality and ${\mathrm{MRR}}$. Besides different study populations and time periods, these different methodological approaches might explain why we estimated higher T2D-free life expectancy. Accordingly, Cunningham et al. [13] found that fixing general mortality rates at levels observed between 1980 and 1989, results in much lower T2D-free life expectancy between 2000 and 2005, compared to using observed mortality rates between 2000 and 2005. They concluded that potential reductions in T2D-free life expectancy due to increases in incidence rates were partly offset by decreases in general mortality rates. This is in line with our finding that T2D-free life expectancy will increase up to the year 2040, even if the incidence rate remains constant.

A similar reasoning holds for the comparison of previous studies estimating ${\mathrm{YLL}}$ associated with T2D. In general, we found lower ${\mathrm{YLL}}$ on the individual level compared to studies from other countries [9, 25–29]. Assuming continuing decreases in T2D-associated excess mortality, we estimated that women and men aged 40 years in 2015 will lose 1.6 years and 2.7 years, respectively. Other studies estimated ${\mathrm{YLL}}$s between 3.5 years among Swedish men aged 40 years in 2013 [29] and 8.5 years among Danish men aged 40 between 1995 and 2008 [9]. This range covers our results in the scenario assuming constant ${\mathrm{MRR}}$ (scenario A). Hence, the lower ${\mathrm{YLL}}$ in our base case scenario are probably mainly caused by assuming continuing decreases in general as well as excess mortality.

Due to different population sizes, our population-wide estimates of ${\mathrm{YLL}}$ cannot be compared to other countries. The GBD provides country-specific ${\mathrm{YLL}}$s on the population level. However, the method is based on T2D-related deaths documented in death certificates and does not consider prevalent cases. Using this method, it was estimated that 256,217 years were lost among deaths with T2D as the documented cause of death in 2015 [10]. As expected, this is far below our estimate of 10.8 million ${\mathrm{YLL}}$ associated with T2D, since our approach summarizes all individual differences in life expectancies between people with prevalent T2D compared to same-aged persons without T2D.

### Implications for public health

Given assumed trends in general mortality and excess mortality associated with T2D, the results suggest a substantial improvement of ${\mathrm{YLL}}$ associated with T2D and T2D-free life expectancy in the German population between 2015 and 2040. This should motivate further efforts to lower the incidence and excess mortality of T2D. This is particularly important, because we also found that increases in incidence and a sustained high T2D-associated excess mortality would lead to substantial increases in disease burden.

The results may be used to inform about the impact of future efforts in treating and preventing T2D. In this regard, two different mechanisms could be the target of interventions. Measures generally known as ‘primary prevention’ aim to prevent disease and thus target the incidence rate [30]. Given an effective measure of primary prevention, this would impact T2D-free life expectancy and ${\mathrm{YLL}}$ on the population level, but would not improve ${\mathrm{YLL}}$ in individuals with T2D. Examples for primary prevention include taxes on unhealthy products (e.g. sugar-sweetened beverages), food labeling and setting based approaches that support healthy food choices [31, 32]. With regard to food labeling, the nutriscore was recently introduced to Germany [33].

Contrary, tertiary prevention strategies aim at reducing the risk of complications among those with the disease [30], for instance by optimizing glucose control or screening for early stages of complications. One example for tertiary prevention in Germany are disease management programs for diabetes, which are structured models of diabetes care provided by health care institutions in cooperation with health insurances. This type of prevention may improve excess mortality of T2D and thus mainly improve ${\mathrm{YLL}}$ on the individual and population level, but not the T2D-free life expectancy.

In light of past decreases in excess mortality observed in other countries, health care systems were rather successful with regard to tertiary prevention [6, 7]. In contrast, heterogeneous trends in the incidence rate across countries do not suggest consistent improvements with regard to primary prevention [23, 34–37]. One might conclude that enhanced efforts of primary prevention are warranted, both to increase T2D-free life expectancy and decrease population-wide ${\mathrm{YLL}}$ associated with T2D.

Another important implication is that the methods used to estimate ${\mathrm{YLL}}$ and T2D-free life expectancy strongly influence the results. Here, we argue that these measures of disease burden involve assumption on future trends by definition, since they rely on life expectancy. Hence, incorporating best available evidence on future trends of mortality and incidence rates may yield more valuable estimates to inform policy than assuming currently observed rates [8].

### Strengths and limitations

This is the first study that projects the future burden of T2D in terms of YLL associated with T2D and T2D-free life expectancy based on data comprising approximately 90% of the population in Germany. Previous studies in the German context reported only one of these measures and did not project future trends in the disease burden associated with T2D. The estimation of ${\mathrm{YLL}}$ and T2D-free life expectancy in this study was based on projected incidence and mortality rates. Compared to assuming currently observed rates, this may be a more valid approximation because these measures are a function of life expectancy which inherently involve future mortality rates. This is particularly relevant when assessing time trends in ${\mathrm{YLL}}$ and T2D-free life expectancy. For instance, Muschik et al. [12] found a decrease in T2D-free life expectancy between 2005 and 2014, using period life tables. It is not clear if these decreases are due to increases in incidence or due to ignoring future trends in mortality. Our results suggest, that T2D-free life expectancy increases even if the incidence rate remains constant, because of decreasing overall mortality. Hence, in the case of Muschik et al., one might conclude that the population will have shorter life time without T2D, without knowing if this would also be concluded if future trends in mortality were incorporated.

As a drawback, we had to rely on more or less speculative assumptions on future trends. This may be particularly problematic given the long projection over 85 years up to the year 2100. While future mortality rates of the general population are based on sound data dating back to 1871, assumptions on trends in the incidence rate were rather arbitrary. In fact, the input data for the incidence originates from 2010, which is quite dated. Given heterogeneous trends in Europe [23, 34–37], we were not able to establish a most plausible trend in incidence. Hence, we assumed constant incidence rates from 2010 onwards in the base case scenario and additionally included scenarios with varying time trends to address the lack of input data. With regard to trends in ${\mathrm{MRR}}$, we mostly relied on data outside of Germany. In contrast to trends in the incidence rate, there is consistent evidence from several high income countries that the ${\mathrm{MRR}}$ decreased over the last decades. Hence, we assumed a continued decrease in the base case scenario and compared the results to a scenario with constant ${\mathrm{MRR}}$. Some studies suggest that the trends in ${\mathrm{MRR}}$ differ between age groups [38]. For instance, in Australia, it was estimated that decreases in the ${\mathrm{MRR}}$ only occurred among people aged ≥ 80 years [39]. However, due to the lack of data in Germany, we assumed the same trend in ${\mathrm{MRR}}$ for all age groups. Technically, age-specific trends in ${\mathrm{MRR}}$ could be incorporated into the projection model.

Another debatable issue in our analysis is that in order to calculate ${\mathrm{YLL}}$, we assumed that a person without T2D at a given age and in a given year will not develop T2D after that year. Of course, this is an unrealistic assumption. Nevertheless, it provides valuable information, since ${\mathrm{YLL}}$ can then be interpreted as the potential life years that would be gained, if excess mortality associated with T2D was non-existent. The model we used would allow to incorporate the more realistic setting, in which persons could develop T2D after a given year. However, the resulting ${\mathrm{YLL}}$ estimate would be lower in scenarios with increasing incidence rates, because persons without T2D in a given year would be more likely to develop T2D and subsequently experience higher mortality rates. Measures of disease burden that indicate lower disease burden when the incidence rate increases are not useful to inform public health.

Finally, our study only considers diagnosed T2D, since we used data from statutory health insurance. Hence, our results do not consider people with undiagnosed T2D and those who did not seek health care in a given year. Furthermore, people in private health insurance are not included, which can be considered a minor issue, given that approximately 90% of the population in Germany is in statutory health insurance.

## Conclusions

Given an assumed decrease in mortality and no increases in T2D incidence, the mortality burden in people with T2D will strongly decrease on the individual as well as on the population level. In addition, the lifetime without T2D is likely to increase. However, due to improved survival, people will also have longer lives with T2D which will further shift the disease burden from mortality to morbidity. The projected improvements in disease burden strongly depend on future improvements of excess mortality associated with T2D and the incidence of T2D, which should motivate increased efforts of primary and tertiary prevention.

## Supplementary Information

**Additional file 1.** Figures illustrating the input data. This additional file includes figures for the prevalence and incidence of type 2 diabetes, the mortality rate ratio associated with type 2 diabetes and the mortality rate of the general population.

**Additional file 2.** Monte Carlo simulation to evaluate the impact of sampling error in the input data on the results from the projection model. This additional file describes the methods and results to evaluate the uncertainty in the projection model due to sampling error of the input data.

**Additional file 3.** Estimation of the survival functions. This additional file specifies the methods used to estimate the survival functions for people with and without type 2 diabetes.

## Data Availability

All data analysed during this study are publicly available from the sources referenced in the “Methods” section.

## Abbreviations

GBD: Global Burden of Disease Study
MRR: Mortality rate ratio
YLL: Years of life lost
T2D: Type 2 diabetes
