# Supplementary material for: Diabetes free life expectancy and years of life lost associated with type 2 diabetes: projected trends in Germany between 2015 and 2040
Source: Popul Health Metr. 2021 Oct 11;19:38. doi: 10.1186/s12963-021-00266-z (PMC8507142; doi:10.1186/s12963-021-00266-z)
Supplement: Supplementary file 1 — Additional file 1. Figures illustrating the input data. This additional file includes figures for the prevalence and incidence of type 2 diabetes, the mortality rate ratio associated with type 2 diabetes and the mortality rate of the general population. [file 12963_2021_266_MOESM1_ESM.pdf]

Diabetes free life expectancy and years of life lost associated with type 2 diabetes: Projected trends in Germany between 2015 and 2040

Input data

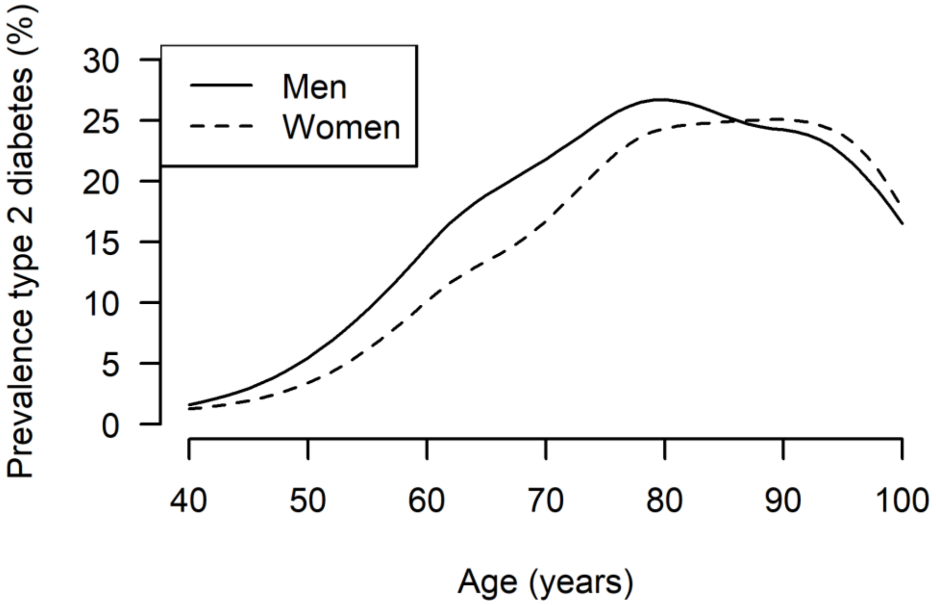

Figure S1: Prevalence of type 2 diabetes in Germany in 2010 based on Tamayo et al. [1].

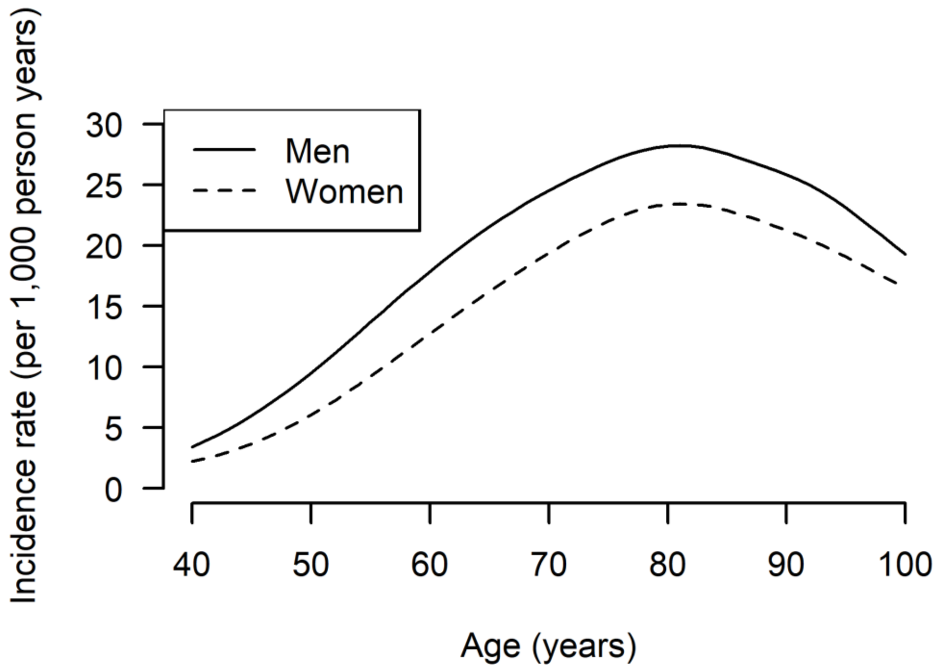

Figure S2: Incidence rate of type 2 diabetes in Germany in 2010 based on Tamayo et al. [1].

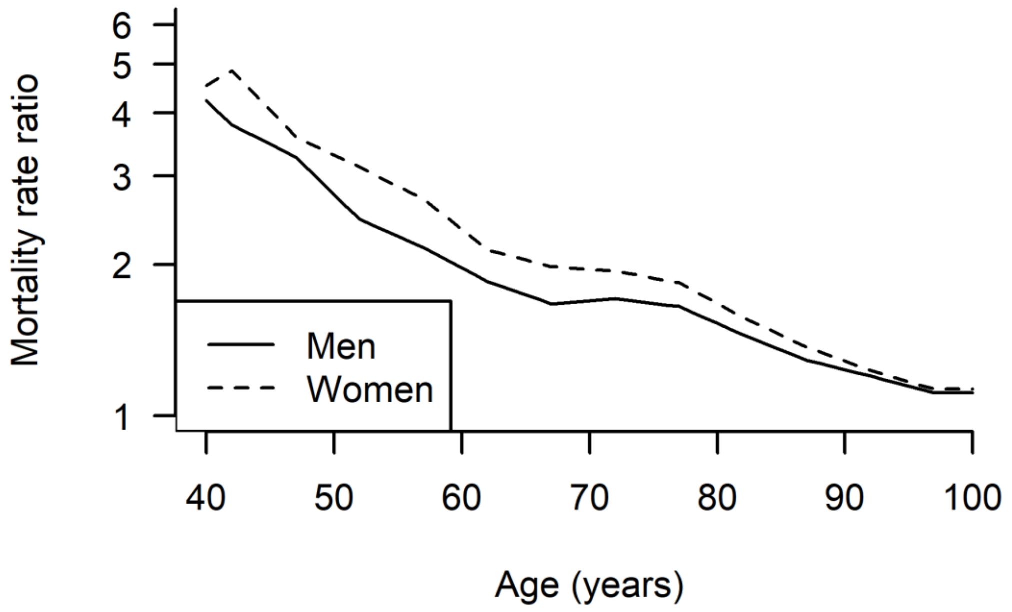

Figure S3: Mortality rate ratio associated with diabetes in Germany in 2013 based on Schmidt et al. [2].

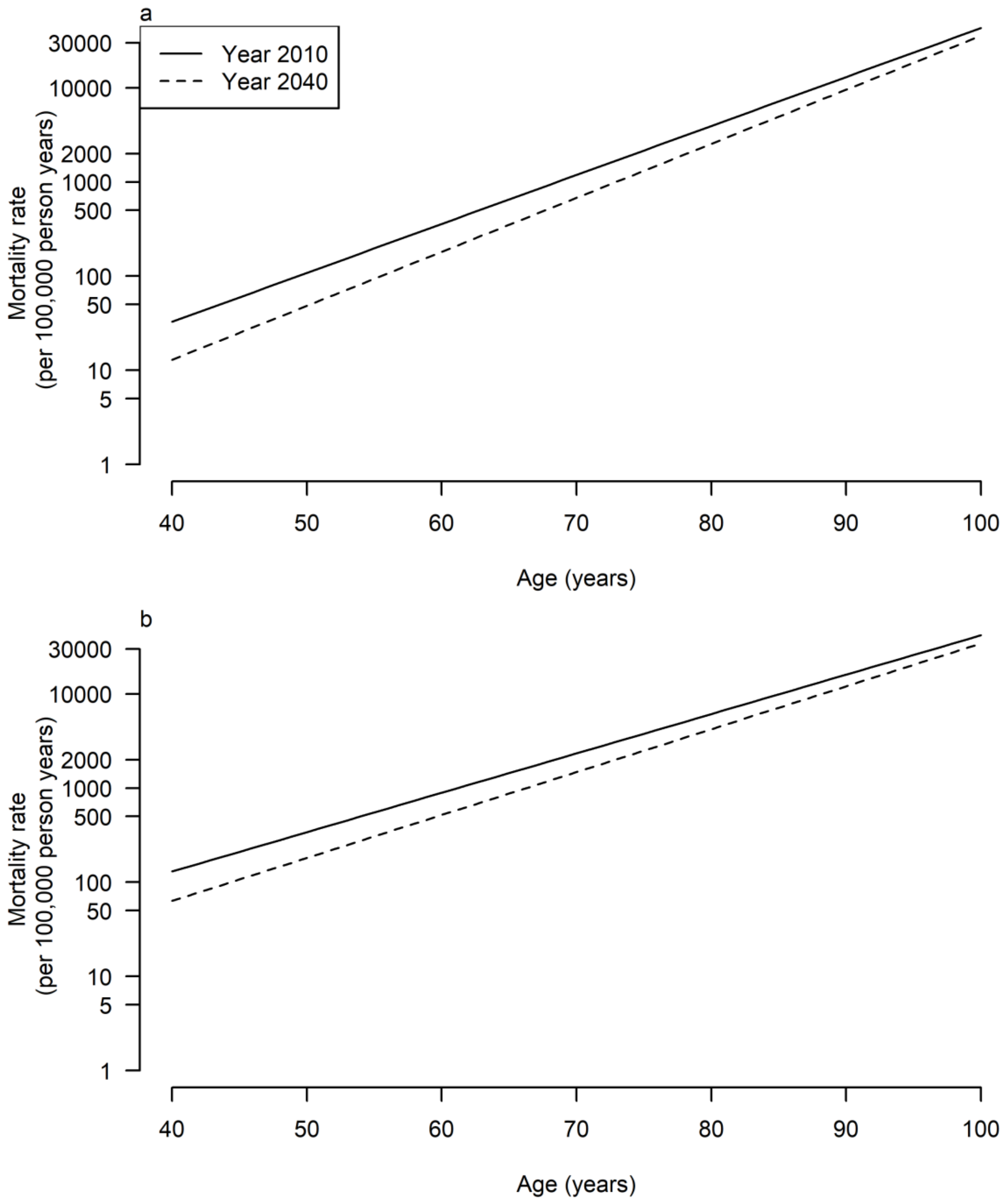

Figure S4: Mortality rate of the general population in Germany among women (a) and men (b) based on the Federal Statistical Office [3].

References

[1] Tamayo T, Brinks R, Hoyer A, Kuß O, Rathmann W (2016) The Prevalence and Incidence of Diabetes in Germany: An Analysis of Statutory Health Insurance Data on 65 Million Individuals From the Years 2009 and 2010. Dtsch Arztebl Int 113: 177-182

[2] Schmidt C, Reitzle L, Heidemann C, et al. (2021) Excess mortality in adults with documented diabetes in Germany: routine data analysis of all insurance claims in Germany 2013–2014. BMJ Open 11: e041508

[3] Statistisches Bundesamt (2015) Bevölkerung Deutschlands bis 2060 - Tabellenband - Ergebnisse der 13. koordinierten Bevölkerungsvorausberechnung. Available from https://www.destatis.de/DE/Publikationen/Thematisch/Bevoelkerung/VorausberechnungBevoelkerung/BevoelkerungDeutschland2060.html, accessed 06 August 2019
