# Supplementary material for: Diabetes free life expectancy and years of life lost associated with type 2 diabetes: projected trends in Germany between 2015 and 2040
Source: Popul Health Metr. 2021 Oct 11;19:38. doi: 10.1186/s12963-021-00266-z (PMC8507142; doi:10.1186/s12963-021-00266-z)
Supplement: Supplementary file 2 — Additional file 2. Monte Carlo simulation to evaluate the impact of sampling error in the input data on the results from the projection model. This additional file describes the methods and results to evaluate the uncertainty in the projection model due to sampling error of the input data. [file 12963_2021_266_MOESM2_ESM.pdf]

Diabetes free life expectancy and years of life lost associated with type 2 diabetes: Projected trends in Germany between 2015 and 2040

Monte Carlo simulation to evaluate the impact of sampling error in the input data on the results from projection model

To evaluate the uncertainty of the results due to sampling error in the input data, we used a Monte Carlo simulation approach. This was done by randomly sampling input values for Equations 1, 2 and 3 in the main text from the distribution of the age-specific prevalence, age-specific incidence and age-specific mortality rate ratio. Using these sampled input values, we proceeded with the projection as described in the main text. This procedure was repeated 1,000 times. From these 1,000 projected estimates of T2D-free life expectancy and YLL, the 2.5^{th} and 97.5^{th} percentiles serve as the lower and upper bound of the 95%-confidence interval. For illustration purposes, the Monte Carlo simulation was only applied to the base case scenario.

Table 1 shows the results of the analysis for the year 2040. As a comparison, the range of results from the alternative projection scenarios in the main text is also shown in Table 1. The comparison shows that the range from the scenario results is substantially wider than the 95%-confidence interval from the Monte Carlo simulation. Hence, in the main text, we do not report 95%-confidence intervals, but instead focus on the variability between the scenarios.

Table 1. Comparison of 95%-confidence interval from Monte Carlo simulation and range of projection scenarios

	Point estimate (base case)	95%-CI	Range of scenarios A,B,C
	**Women**		
**Individual level YLL (years)**	0.7	0.7-0.9	0.7-3.1
**Population level YLL (million years)**	2.77	2.73-3.38	2.56-10.11
**T2D-free life expectancy (years)**	39.1	39.0-39.2	36.4-41.4
	**Men**		
**Individual level YLL (years)**	1.1	1.1-1.4	1.1-4.4
**Population level YLL (million years)**	3.58	3.55-4.50	3.31-12.63
**T2D-free life expectancy (years)**	34.0	33.9-34.2	31.5-36.4

Individual level YLL and T2D-free life expectancy refer to age 40 years. Details on the projection scenarios is available in the main text.
YLL, years of life lost; T2D, type 2 diabetes
