# Supplementary material for: Diabetes free life expectancy and years of life lost associated with type 2 diabetes: projected trends in Germany between 2015 and 2040
Source: Popul Health Metr. 2021 Oct 11;19:38. doi: 10.1186/s12963-021-00266-z (PMC8507142; doi:10.1186/s12963-021-00266-z)
Supplement: Supplementary file 3 — Additional file 3. Estimation of the survival functions. This additional file specifies the methods used to estimate the survival functions for people with and without type 2 diabetes. [file 12963_2021_266_MOESM3_ESM.pdf]

Diabetes free life expectancy and years of life lost associated with type 2 diabetes: Projected trends in Germany between 2015 and 2040

Estimation of the survival functions

For the estimation of HLY and YLL using Eqs. (2) and (3) in the main text, survival functions were needed. Let $j = 0{,}1$ denote the states 'no type 2 diabetes' and 'type 2 diabetes', respectively. Then the survival function was defined as

$$S_j(t,a) = \exp\left(-\sum m_j(t,a)\right)$$

where t is the calendar time, a is the age and m_j is the mortality rate at age a and time t. Furthermore, let $m(t,a) = p(t,a) \times m_1(t,a) + \left(1 - p(t,a)\right) \times m_0(t,a)$ denote the mortality of the general population with $p(t,a)$ the prevalence of type 2 diabetes. The mortality rate in the general population was derived from the life tables of official population projections assuming the Gompertz-Makeham mortality law. The Gompertz-Makeham mortality law states that the mortality rate increases exponentially with age. This assumption is based on theoretical and empirical considerations [1, 2]. In the different scenarios described in the main text, we projected age-specific values for the prevalence and the mortality rate ratio (MRR). Using these projected values, the mortality rate of people without type 2 diabetes can be obtained by

$$m_0(t,a) = \frac{m(t,a)}{1 + p(t,a) \times (MRR(t,a) - 1)}$$

and the mortality rate of people with type 2 diabetes by

$$m_1(t,a) = m_0(t,a) \times MRR$$

The mortality rates m, m_0 and m_1 were used to estimate the survival functions S, S_0 and S_1 for the general population, the population without type 2 diabetes and the population with type 2 diabetes, respectively.

References

[1] Missov TI, Lenart A (2013) Gompertz–Makeham life expectancies: Expressions and applications. Theor Popul Biol 90: 29-35

[2] Carstensen B, Kristensen JK, Ottosen P, Borch-Johnsen K, Steering Group of the National Diabetes Register (2008) The Danish National Diabetes Register: trends in incidence, prevalence and mortality. Diabetologia 51: 2187-2196
